# Development of *Cordyceps javanica* BE01 with enhanced virulence against *Hyphantria cunea* using polyethylene glycol-mediated protoplast transformation

**DOI:** 10.3389/fmicb.2022.972425

**Published:** 2022-09-02

**Authors:** Wenxiu Wang, Yahong Wang, Guangping Dong, Fengmao Chen

**Affiliations:** ^1^Collaborative Innovation Center of Sustainable Forestry in Southern China, College of Forestry, Nanjing Forestry University, Nanjing, China; ^2^Key Laboratory of State Forestry Administration on Pine Wilt Disease Prevention and Control, Hefei, China

**Keywords:** *Cordyceps* *javanica*, transformation system, protease, enhanced virulence, *Hyphantria cunea*

## Abstract

*Cordyceps javanica* has promising application prospects as an entomopathogenic fungus with a wide range of hosts. To enhance the virulence of *C*. *javanica*, a polyethylene glycol (PEG)-mediated protoplast genetic transformation system was constructed. Strains overexpressing the subtilisin-like protease genes *CJPRB* and *CJPRB1* and the tripeptidyl peptidase gene *CJCLN2-1* were constructed with this system, and the effects of these strains on *Hyphantria cunea* were tested. The aminoglycoside G418 was used at 800 μg ml^−1^ to screen the transformants. *C*. *javanica* hyphae were degraded with an enzyme mixture to obtain protoplasts at 1.31 × 10^7^ protoplasts ml^−1^. The transformation of 2 μg of DNA into 1,000 protoplasts was achieved with 20% PEG2000, and after 6 h of recovery, the transformation efficiency was 12.33 ± 1.42 transformants μg^−1^ plasmid. The LT_50_ values of *CJPRB*, *CJPRB1,* and *CJCLN2-1*-overexpressing *C*. *javanica* strains were 1.32-fold, 2.21-fold, and 2.14-fold higher than that of the wild-type (WT) strain, respectively. The three overexpression strains showed no significant differences from the WT strain in terms of colony growth, conidial yield, and conidial germination rate. However, the infection rate of the CJPRB1 strain was faster than that of the WT strain, with infection occurring within 4–5 days. The CJCLN2-1 strain had a significantly higher mortality rate than the WT strain within 4–10 days after infection. A *C*. *javanica* genetic transformation system was successfully constructed for the first time, and an overexpression strain exhibited enhanced virulence to *H*. *cunea* compared with the WT strain.

## Introduction

Entomopathogenic fungi (EPFs) are recognized as important natural regulators of insect pest populations and provide an environmentally friendly alternative to chemical pesticides (Qu and Wang, 2018). Typically, the EPF releases an array of hydrolytic enzymes (proteases, chitinases, and lipases) to achieve cuticle penetration and then reaches the hemocoel through the cuticle, where it absorbs nutrients, releases toxins, destroys host cells, and ultimately kills the host (Thomas and Read, 2007; Schrank and Vainstein, 2010; Valero-Jiménez et al., 2014; Wei et al., 2017). Subtilisin-like proteases (Pr1), a class of cuticle-degrading proteases, have been implicated as virulence factors with potential applications in the engineering of genetically modified fungi for use against insects, possibly enabling hyphal invasion into the insect hemocoel through cuticle infection (Gao et al., 2020). Previous studies have shown that engineered *Beauveria bassiana* and *Metarhizium anisopliae* strains overexpressing Pr1 exhibited LT_50_ (time to kill 50% of *Myzus persicae* and *Manduca sexta,* respectively) values that were decreased by 12.5% and 25% compared with those of the respective wild-type (WT) strains (St Leger et al., 1996; Fang et al., 2009). Thus, these studies showed that genetic modification can produce strains with faster kill rates. In addition, recent studies have shown that tripeptidyl peptidase I (CLN2), which has been widely studied in the context of human disease, may play a role in the infection process of EPFs. Specifically, CLN2 is upregulated during infection and secreted into the hemolymph in insects (Tartar and Boucias, 2004).

*Cordyceps javanica* (formerly known as *Isaria javanica*), belonging to the genus *Cordyceps* (Ascomycota, Sordariomycetes, Hypocreales, Cordycipitaceae, and *Cordyceps*) (Kepler et al., 2017), is an important EPF with a broad host range, infecting insects from 12 genera in 3 orders (Lepidoptera, Hemiptera, and Thysanoptera) (Cabanillas and Jones, 2009; Ren and Hu, 2009; Shimazu and Takatsuka, 2010; Chen et al., 2014; Hu, 2014; Gallou et al., 2016; Lin et al., 2019; Wang et al., 2019). In our previous research, an isolate of *C*. *javanica* BE01 exhibited high virulence against the widespread foliar-feeding pest *Hyphantria cunea* (Drury) (Wang et al., 2019). To enhance the virulence of *C*. *javanica* through genetic modification, further knowledge of the mechanism used by *C*. *javanica* to infect *H*. *cunea* is necessary. Thus, it is necessary to establish an efficient transformation system and construct genetically modified strains to study the functions of virulence-related genes. The first step in establishing the transformation system is to determine the appropriate dominant selection marker. Among selection markers, hygromycin B, Geneticin (G418), and phleomycin are widely used for the establishment of fungal transformation systems (Campbell et al., 2002; Shao et al., 2015; Nai et al., 2017; He et al., 2020). At present, the genetic transformation systems of fungi in the genus *Cordyceps* mainly include the *Agrobacterium*-mediated genetic transformation system and the polyethylene glycol (PEG)-mediated protoplast genetic transformation system (Huang et al., 2016; Lou et al., 2019). PEG-mediated protoplast genetic transformation has been successfully applied to many species of fungi (Wei et al., 2010; Zhang et al., 2022). Protoplast preparation is the first step for the successful PEG-mediated genetic transformation of protoplasts. In addition, there are many factors that affect the efficiency of the protoplast transformation protocol, including the molecular weight and concentration of PEG, protoplast recovery time, and plasmid concentration (Li et al., 2017; Díaz et al., 2019; Amalamol et al., 2022).

The objective of this study was to establish a PEG-mediated genetic transformation system for *C*. *javanica* and to identify the factors affecting the transformation efficiency. In addition, we further investigated whether overexpression of the *C*. *javanica* subtilisin-like proteases *CJPRB* and *CJPRB1* and the tripeptidyl peptidase *CJCLN2-1* could enhance the virulence of *C*. *javanica* against *H*. *cunea*.

## Materials and methods

### Strain, culture conditions, and plasmid

The strain *C*. *javanica* BE01 was isolated from *H*. *cunea* larvae (Wang et al., 2019) and routinely maintained on sabouraud dextrose agar medium with yeast extract (SDAY) (Gallou et al., 2016). BE01 was grown on SDAY at 25°C for 5 days. Then, the young mycelia were removed for shake flask cultivation.

The plasmid PYF11-GFP used in the experiment was stored in the Pathology Laboratory of Nanjing Forestry University. This plasmid includes an RP27 promoter, a green fluorescent protein gene (*gfp*), a TrpC promoter, a *neo* gene, and a TrpC terminator. The *neo* gene encodes a neomycin phosphotransferase that mediates resistance to G418 (a broad-spectrum aminoglycoside antibiotic).

The plasmids PYF11-CJPRB, PYF11-CJPRB1, and PYF11-CJCLN2-1 were constructed. The identification and phylogenetic tree of the subtilisin-like proteases CJPRB and CJPRB1 and tripeptidyl peptidase CJCLN2-1 (GenBank: URX52600.1, URX52601.1, URX52602.1) are shown in Supplementary Table 1 and Supplementary Figure 1. The coding sequences of *CJPRB*, *CJPRB1,* and *CJCLN2-1* (GenBank: OM468894, OM468895, and OM468896) were cloned from *C*. *javanica* strain BE01 with the primer pairs CJPRB-F/R, CJPRB1-F/R, and CJCLN2-1-F/R, respectively (Supplementary Table 2). Then, the *CJPRB*, *CJPRB1,* and *CJCLN2-1* fragments were ligated into XhoI-cut PYF11-GFP to generate PYF11-CJPRB, PYF11-CJPRB1, and PYF11-CJCLN2-1, respectively. Homologous DNA recombination was performed using a ClonExpress Ultra One Step Cloning Kit (Vazyme Biotech Co., Ltd).

### Antibiotic resistance test of *Cordyceps* *javanica* BE01

BE01 conidia (1 × 10^2^ conidia ml^−1^) were added to 15 ml of SDAY supplemented with Geneticin and different concentrations of the aminoglycoside G418 (0, 100, 200, 300, 400, 500, 600, 700, 800, 900, or 1,000 μg ml^−1^). Each mixture was poured into a Petri dish and incubated at 25°C for 7 days, and the growth of BE01 was observed. Antibiotic concentrations that completely inhibited fungal growth were used for fungal genetic transformation.

### Polyethylene-glycol-mediated transformation of *Cordyceps* *javanica* BE01

#### Protoplast preparation

BE01 was cultured in SDY in a shake flask at 25°C and 110 rpm for 3 days, and then a single-layer Miracloth filter was used to collect mycelia. The mycelia were washed three times with sterile distilled water and then three times with 0.7 M NaCl. In parallel, the enzyme mixture (3 mg ml^−1^ lysing enzyme (Sigma), 7 mg ml^−1^ driselase (Sigma), and 0.5 mg ml^−1^ chitinase (Sigma) added in 30 ml of 0.7 M NaCl solution) was prepared, and filtered through a 0.22 μm filter. The washed mycelia were added to the filtered enzyme mixture, and the mixture was incubated at 25°C and 60 rpm for 3 h. Then, the protoplasts were collected by filtration through a double-layer Miracloth filter. The filtrate containing protoplasts was centrifuged at 2000 rpm for 8 min at 4°C, and the supernatant was discarded. The protoplasts were washed with 0.7 M NaCl solution and then resuspended in STC buffer (sorbitol, 145.74 g; Tris–HCl, 6.06 g; CaCl_2,_ 5.55 g; 1,000 ml of sterile distilled water; pH 8.0). The initial recorded concentration was 1.31 × 10^7^ protoplasts ml^−1^, which was adjusted to 1.0 × 10^4^ protoplasts ml^−1^.

#### Polyethylene glycol/CaCl_2_-mediated fungal transformation

Plasmid (2, 5, or 10 μg) was added to 100 μl of protoplasts in STC buffer (1.0 × 10^4^ protoplasts ml^−1^), and the mixture was incubated at room temperature for 30 min. Then, 1 ml of PTC solution (10–60% PEG2000/4000/6000 in STC buffer) was added, and the mixture was mixed well and kept at room temperature for 20 min. Each mixture was then mixed with 3 ml of SDY and incubated at 25°C and 60 rpm for 0, 6, 12, or 24 h. Then, each mixture was added to freshly autoclaved SDAY (cooled to 50°C) with 800 μg ml^−1^ G418, and the medium was mixed and poured into a Petri dish. After 7 days of incubation at 25°C, transformants could be observed.

### Transformant screening

The positive transformants were subcultured on nonselective SDAY medium and then cultured on selective medium (supplemented with the antibiotic G418) to reveal the resistance phenotype, and confirmed transformants were finally obtained according to the method of Zhang et al. (2016). Then, GFP signals of the conidia and hyphae of the transformants were examined under differential interference contrast optics and fluorescence on an optical microscope (AXIO Imager, Carl Zeiss, Microscopy GmbH, Göttingen, Germany) using a 100× objective. The images were photographed digitally with an AxioCam HR R3 camera (Carl Zeiss) using ZEN Blue Lite 2.3 software (10-2016). GFP expression in the transformants transformed with PYF11-GFP was monitored.

To examine the overexpression of the *CJPRB*, *CJPRB1,* and *CJCLN2-1* genes, total RNA was extracted from the mycelia from the SDAY plates. Each RNA sample was reverse transcribed to cDNA using a HiScript II Q RT SuperMix for qPCR (+gDNA wiper) kit (Vazyme Biotech Co., Ltd). qRT–PCR analysis was performed using a SYBR Green Pro Taq HS qPCR kit (Accurate Biotech Co., Ltd) and primer pairs specific for the different genes (Supplementary Table 2) on an ABI Prism 7,900 system. The translation elongation factor 1-alpha (EF-1α) gene (MG 820547) of *C*. *javanica* was amplified as an internal control. The data were analyzed using the comparative threshold cycle (2^−ΔΔCt^) method (Vicente et al., 2015). The expression of *CJPRB*, *CJPRB1,* and *CJCLN2-1* in the overexpression strain was compared with that in the WT strain.

### Mycelial growth, sporulation, and conidial germination

Agar blocks (5 mm diameter) of different strains were inoculated on SDAY plates and grown for 11 days. The colony diameter was measured by the crisscross method every 2 days beginning on the third day, and the colony growth rate and colony diameter were calculated from 5 measurements. To determine the conidial yield, two 5-mm-diameter agar blocks were removed from each plate, placed in 1 ml of 0.1% (v/v) Tween 80, and vortexed well. The conidial yield was counted by a hemocytometer, and the number of conidia mm^−2^ was calculated. To determine the germination rate, 50 μl of conidial suspension (2 × 10^3^ conidial ml^−1^) was deposited on a hydrophobic glass slide; the slide was placed in a Petri dish covered with moist filter paper, and conidial germination was examined by microscopy after 12 h of incubation at 25°C. All treatments were repeated at least three times.

### Insect bioassays

Wild-type BE01 and transformants were grown on sterile cellophane (on the surface of SDAY medium) at 25°C for 15 days. The cellophane was then individually peeled off from each strain, and the conidia were eluted with 0.1% (v/v) aqueous Tween 80, followed by filtration through sterile cotton to remove any hyphae (Wang et al., 2019). Conidia were counted with a hemocytometer, and the concentration was adjusted to 1 × 10^6^ conidia ml^−1^. The third instar larvae were immersed in conidial suspension (1 × 10^6^ conidia ml^−1^) for 10 s and then placed on sterile filter paper to absorb excess suspension. The control groups were immersed in only 0.1% (v/v) aqueous Tween 80. Larvae were incubated for 15 days at 25 ± 2°C and 65 ± 5% RH with a 12:12 h L:D photoperiod. The poplar leaves used to feed *H*. *cunea* larvae were changed daily. Dead *H*. *cunea* larvae were placed in an empty Petri dish containing sterile water-soaked filter paper and incubated at 25 ± 2°C for observation, and the larvae covered with hyphae were recorded as dead. The dead larvae were counted daily, and the mortality induced by each strain was calculated after 15 days. The median mortality time (LT_50_) of the tested insects was calculated. There were 3 replicates per treatment, with 10 insects per replicate.

### Statistical analysis

Statistical analysis of fungal transformants, fungal growth, sporulation, and spore germination data was performed using one-way analysis of variance (ANOVA) in SPSS v.20 (IBM, Armonk, NY, United States). Statistical analysis of the mortality data was performed using the general linear model (GLM) in SPSS v.20 (IBM, Armonk, NY, United States). The LT_50_ was estimated by probit analysis of the time-mortality trend in each group. The significant differences between treatments were determined by multiple pairwise comparisons using Duncan’s multiple range test and Student’s *t-test*. Statistical significance was set at *p* < 0.05. The data are presented as the mean ± SE.

## Results

### Sensitivity of *Cordyceps* *javanica* BE01 to different concentrations of the aminoglycoside G418

**Figure 1:**
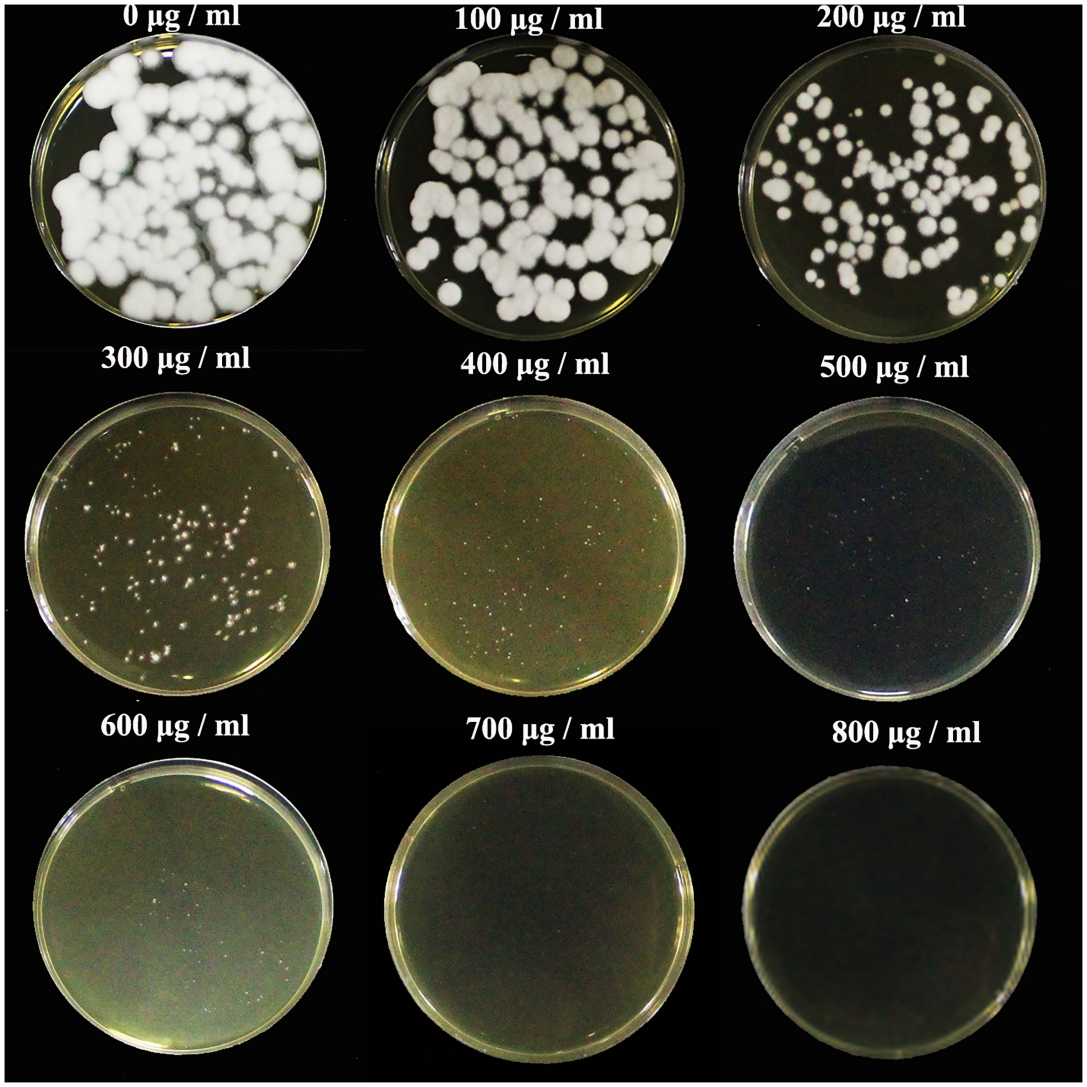
Sensitivity of *Cordyceps* *javanica* BE01 protoplasts to G418. SDAY medium was supplemented with different concentrations of G418 (0–800 μg ml^−1^), and samples were incubated for 7 days at 25°C.

The sensitivity of *C*. *javanica* BE01 to G418 was tested before transformation experiments. G418 resistance was chosen as a marker due to its successful application in fungal molecular genetics. We found that *C*. *javanica* BE01 did not grow at all in SDAY medium containing 800 μg ml^−1^ G418 (Figure 1), so a concentration of 800 μg ml^−1^ as the minimal inhibitory concentration was selected for transformant screening. The entire experiment was repeated three times.

### Influence of PEG molecular weight and concentration on the transformation efficiency of *Cordyceps* *javanica* BE01

**Figure 2:**
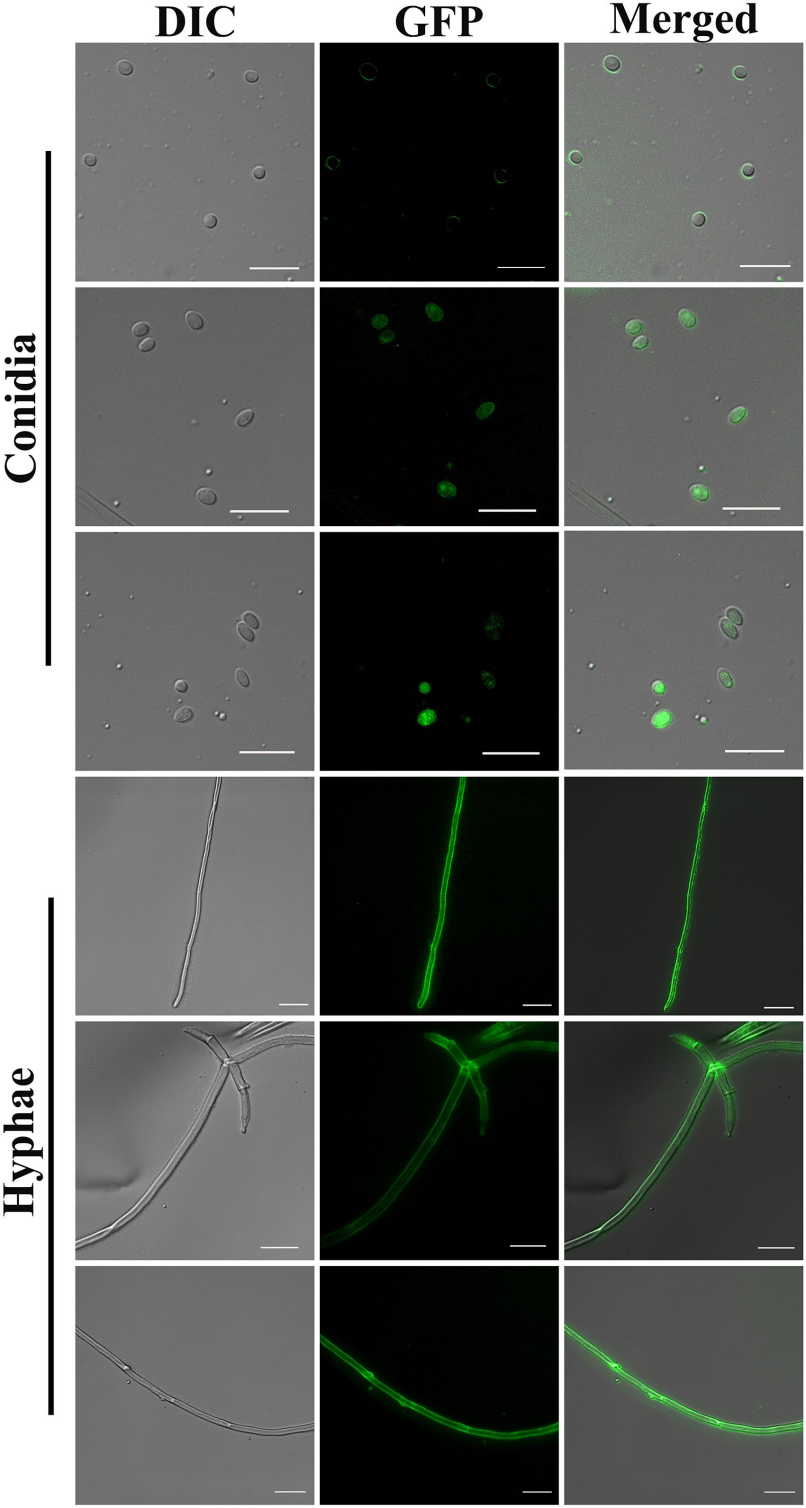
Conidia and hyphae of positive transformants under differential interference contrast and fluorescence microscopy (100× objective). Bar = 10 μm.

The positive transformants were observed to emit green fluorescence under a fluorescence microscope (Figure 2). The results showed that both the molecular weight and concentration of PEG had significant effects on the conversion efficiency of BE01 (*F*_2, 14_ = 5.456, *p* < 0.05; *F*_5, 14_ = 4.261, *p* < 0.05). The effects of different concentrations of PEG2000 on the conversion efficiency of BE01 were significantly different (*F*_3, 8_ = 7.066, *p* < 0.05). The transformation efficiency of BE01 mediated by 20% PEG2000 was significantly higher than that of other concentrations of PEG2000, and 14.00 ± 3.79 transformants were obtained, with a transformation efficiency of 7.00 ± 1.89 transformants μg^−1^ plasmid (Table 1). In addition, the effects of different concentrations of PEG4000 and PEG6000 on the conversion efficiency of BE01 were not significantly different (*F*_3, 8_ = 1.153, *p* > 0.05; *F*_4, 10_ = 0.286, *p* > 0.05). We also found that the conversion efficiency of the same concentration of PEG with different molecular weights did not differ significantly, except at a concentration of 20% (10%: *F*_2, 6_ = 0.333, *p* > 0.05; 20%: *F*_3, 8_ = 9.760, *p* < 0.05: 30%: *F*_2, 6_ = 4.159, *p* > 0.05; 40%: *t* = 1.061, df = 4, *p* > 0.05). The transformation efficiency with 20% PEG2000 was significantly higher than that with 20% PEG4000 and PEG6000, and the transformation efficiency with 30% PEG2000 was significantly higher than that with PEG4000 at the same concentration, reaching 5.67 ± 1.45 transformants; the overall transformation efficiency was 2.83 ± 0.73 transformants μg^−1^ plasmid (Table 1). Based on these findings, 20% PEG2000 was selected for subsequent experiments.

**Table 1:** Effects of different molecular weights and concentrations of polyethylene glycol (PEG) on the number of transformants (mean ± SE).

PEG concentration (m/v)	Number of transformants			*p*-value^a^
	PEG2000	PEG4000	PEG6000	
10%	1.33 ± 0.67	0.67 ± 0.33	1.33 ± 0.88	*p* > 0.05
20%	14.00 ± 3.79	2.33 ± 1.20	0.67 ± 0.67	*p* < 0.05
30%	5.67 ± 1.45	1.00 ± 1.00	1.33 ± 1.33	*p* > 0.05
40%	–	0.33 ± 0.33	1.33 ± 0.88	*p* > 0.05
50%	1.67 ± 1.67	–	–	–
60%	–	–	0.33 ± 0.33	–
*p*-value^b^	*p* < 0.05	*p* > 0.05	*p* > 0.05	–

a Significance of the difference between the means in each row (*p* < 0.05 indicates a significant difference; 10–30%: Duncan’s multiple range test; 40%: Student’s *t*-test).

b Significance of the difference between the means in each column (*p* < 0.05 indicates a significant difference, Duncan’s multiple range test).

### Effects of protoplast recovery time and plasmid DNA amount on the transformation efficiency of *Cordyceps* *javanica* BE01

The transformation efficiency of *C*. *javanica* BE01 was significantly different among treatments with different recovery times (*F*_3, 8_ = 9.481, *p* < 0.05). The number of transformants obtained after 6 h of protoplast recovery was significantly higher than that after the other recovery times, reaching 24.67 ± 2.85 transformants, and the overall transformation efficiency was 12.33 ± 1.42 transformants μg^−1^ plasmid (Table 2). In addition, the transformation efficiency (7.50 ± 1.80 transformants μg^−1^ plasmid) of protoplasts recovered at 0 h was not significantly different from that of protoplasts recovered at 24 h (6.17 ± 0.60 transformants μg^−1^ plasmid) but was significantly higher than that of protoplasts recovered at 12 h (2.50 ± 1.15 transformants μg^−1^ plasmid) (Table 2). The numbers of transformants obtained by transformation of different amounts of plasma DNA into *C*. *javanica* BE01 protoplasts were significantly different (*F*_2, 6_ = 27.957, *p* < 0.05). The efficiency of transformation with 5 μg of plasmid was the highest, with a value of 4.07 ± 0.47 transformants μg^−1^ plasmid obtained, which was significantly higher than the transformation efficiencies obtained with 2 μg and 10 μg plasmid (Table 3).

**Table 2:** Effect of time of recovery on the number of transformants (mean ± SE).

Recovery time (h)	Number of transformants
0	15.00 ± 3.61
6	24.67 ± 2.85
12	5.00 ± 2.31
24	12.33 ± 1.20
*p*-value^a^	*p* < 0.05

a Significance of the difference between the means in each column (*p* < 0.05 indicates a significant difference, Duncan’s multiple range test).

**Table 3:** The effect of plasmid amount on the number of transformants (mean ± SE).

Plasmid DNA (μg)	Number of transformants
2	4.00 ± 1.15
5	20.33 ± 2.33
10	8.00 ± 1.00
*p*-value^a^	*p* < 0.05

a The significance of the difference between the means in each column (*p* < 0.05 indicated a significant difference, Duncan’s multiple range test).

### Growth, sporulation, and conidial germination of overexpression strains

**Figure 3:**
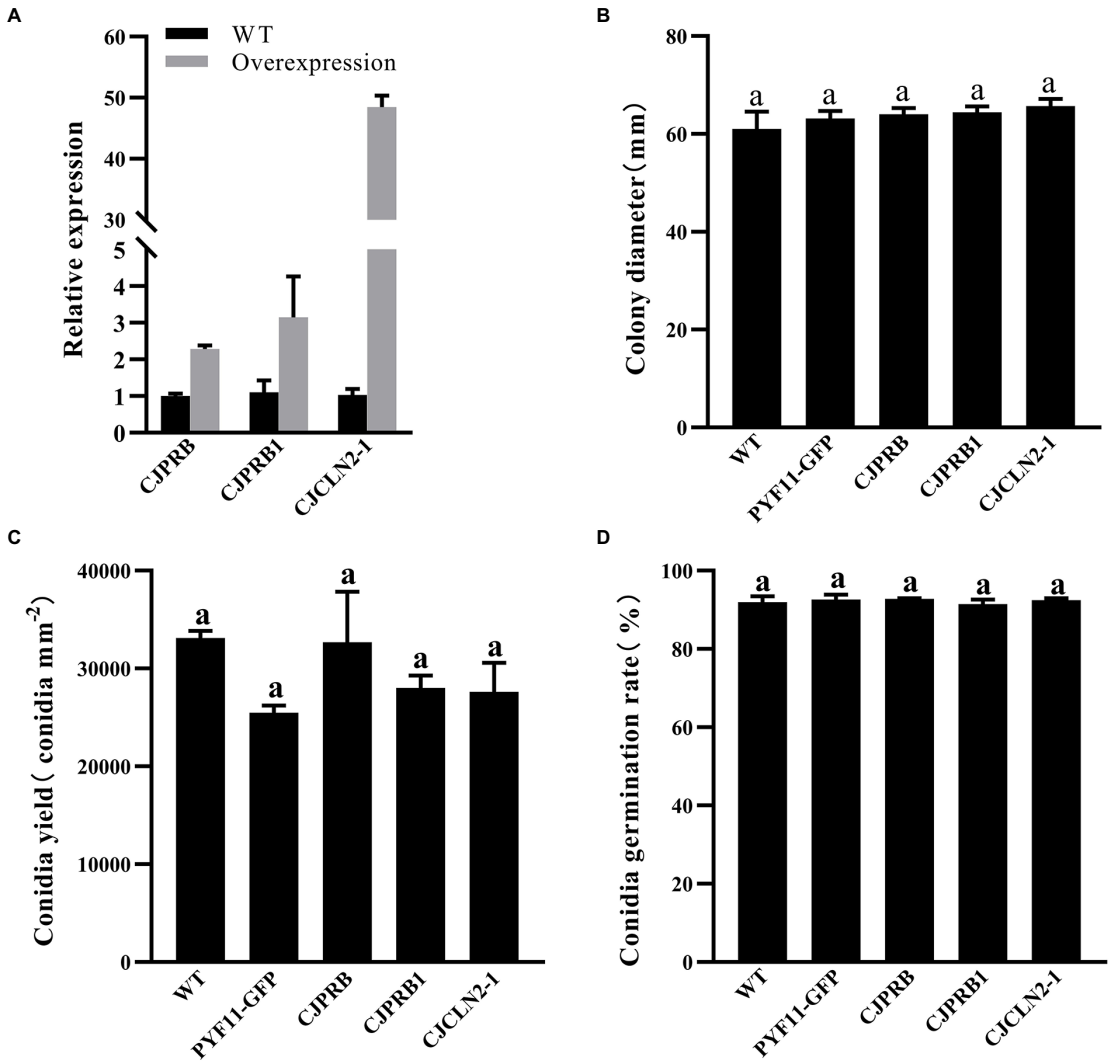
qRT–PCR and phenotype verification of CJPRB-, CJPRB1-, and CJCLN2-1-overexpressing BE01 strains. **(A)** Validation of overexpression strains by qRT–PCR. **(B–D)** Colony diameter, conidial production, and conidial germination rate of wild-type, PYF11-GFP-transformed, and overexpression strains of BE01 (mean ± SE). A common lowercase letter above the error bar is not significantly different (*p*>0.05, Duncan’s multiple range test).

The qRT–PCR results showed that the expression of *CJPRB*, *CJPRB1*, and *CJCLN2-1* in the overexpression strains was upregulated 2.28 ± 0.10-, 3.15 ± 1.12-, and 48.44 ± 1.93-fold, respectively, compared with that in the WT strain, indicating that strains overexpressing the above three genes were successfully constructed (Figure 3A). The colony diameter, conidial yield and conidial germination rate of these three overexpression strains were not significantly different from those of the WT or PYF11-GFP transformed strains (colony diameter: *F*_4, 15_ = 0.768, *p* > 0.05; conidial yield: *F*_4, 10_ = 1.479, *p* > 0.05; conidial germination rate: *F*_4, 10_ = 0.283, *p* > 0.05) (Figures 3B–D). In addition, the abovementioned overexpression strains had no significant difference in growth rate from the WT and PYF11-GFP transformed strains (Table 4).

**Table 4:** Regression equations of colony growth of different strains of *C*. *javanica* BE01 on SDAY medium (mean ± SE).

Strain	Growth regression equation	Growth rate (mm/d)	Correlation coefficient
WT	*y* = 4.264*x* + 13.658	4.264 ± 0.317	0.910
PYF11-GFP	*y* = 4.501*x* + 13.607	4.501 ± 0.151	0.980
CJCLN2-1	*y* = 4.768*x* + 13.05	4.768 ± 0.144	0.984
CJPRB1	*y* = 4.62*x* + 13.863	4.62 ± 0.159	0.979
CJPRB	*y* = 4.54*x* + 14.381	4.54 ± 0.122	0.987

### Contribution of *CJPRB, CJPRB1, and CJCLN2-1* to the Virulence of *Cordyceps* *javanica* BE01

**Figure 4:**
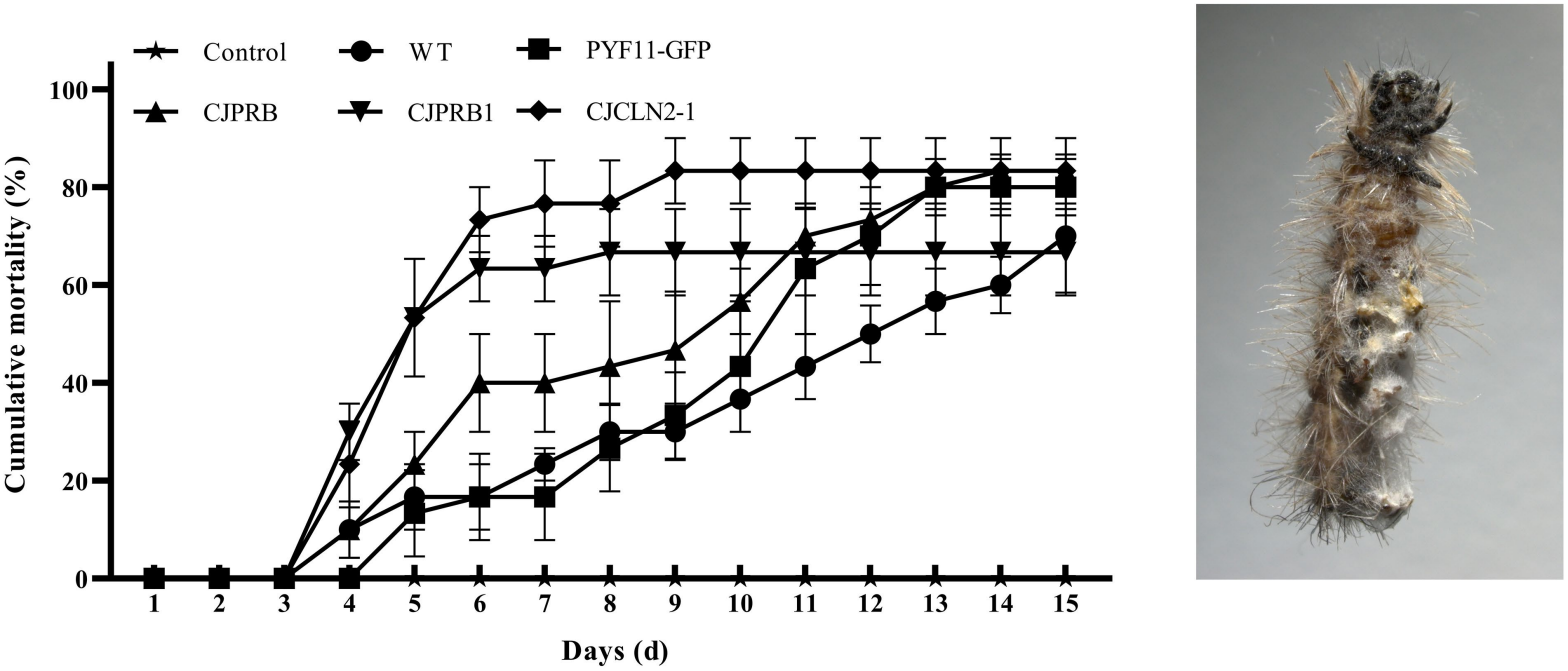
Cumulative mortality (mean ± SE) of *H*. *cunea* larvae in response to different types of *C*. *javanica* BE01 strains over 15 days. Cumulative mortality (mean ± SE) of *H*. *cunea* larvae (left) and dead larvae covered with mycelia (right).

*H*. *cunea* larvae were treated with different strains overexpressing BE01 for 15 days, and the overall induced mortality rates did not differ significantly among the strains (*F*_4, 10_ = 1.019, *p* > 0.05) (Table 5 and Figure 4). However, the mortality induced between days 4 and 10 of infection differed significantly among strains (*F*_4, 70_ = 40.326, *p* < 0.05) (Figure 4). In particular, *CJCLN2-1-and CJPRB1*-overexpressing strains caused significantly higher mortality than the WT and PYF11-GFP-transformed strains within 4–10 days and 4–5 days of infection, respectively (*F*_2, 42_ = 74.353, *p* < 0.05; *F*_2, 15_ = 9.868, *p* < 0.05) (Figure 4). In addition, there were significant differences in LT_50_ among different BE01 strains (*F*_4, 10_ = 12.931, *p* < 0.05) (Table 5). The LT_50_ values of *CJCLN2-1* and *CJPRB1*-overexpressing strains were significantly lower than those of the WT and *CJPRB*-overexpressing strains, with values of 5.18 ± 0.80 days and 5.01 ± 0.57 days, respectively (Table 5).

**Table 5:** Insect bioassay of *C*. *javanica* wild-type transformant strains (mean ± SE).

Strain	Mortality rate (%)	LT_50_ (d)
Control	0	0
WT	70.00 ± 11.55	11.08 ± 0.52
PYF11-GFP	80.00 ± 5.77	9.89 ± 1.00
CJPRB	83.33 ± 3.33	8.39 ± 0.83
CJPRB1	66.67 ± 8.82	5.01 ± 0.57
CJCLN2-1	83.33 ± 6.67	5.18 ± 0.80
*p*-value^a^	*p* > 0.05	*p* < 0.05

a Significance of the difference between the means in each column (*p* < 0.05 indicates a significant difference, Duncan’s multiple range test).

LT_50_, median mortality time of the tested insects.

## Discussion

The establishment of genetic transformation systems for EPFs provides a basis for studying their pathogenic mechanisms and constructing genetically modified strains. The first step for constructing a genetic transformation system is to choose an appropriate dominant selection marker. Next, the minimum inhibitory concentration of the selection agent required to prevent the growth of nontransformed cells and selection of false transformants must be determined (Lim et al., 2021). The aminoglycoside G418 is widely used as a selection marker in transformation systems in fungi, plants, and mammals (Itaya et al., 2018; He et al., 2020; Huang et al., 2020). Therefore, in our study, G418 was chosen as the selection marker, and 800 μg ml^−1^ G418 was determined to be the lowest concentration that could reliably inhibit the growth of *C*. *javanica* BE01.

The PEG-mediated protoplast transformation method is the most commonly used method for the genetic transformation of filamentous fungi. This method consists of three main steps: protoplast acquisition, uptake of transforming plasmids, and growth of transformants on selective media (Liu and Friesen, 2012). In these protocols, cell wall-degrading enzymes (CWDEs) (mainly cellulase, Driselase from *Trichoderma* sp., lysing enzyme, 1,3-glucanase, and chitinase) are used to degrade the cell wall of fungal hyphae or germinated spores to successfully obtain high concentrations of protoplasts, which is a prerequisite for PEG-mediated protoplast transformation (Wei et al., 2010; Liu and Friesen, 2012; Wang et al., 2018; Lim et al., 2021; Liu et al., 2021).

Shimizu et al. found that lysis of *Paecilomyces fumosoroseus* mycelia for 3 h with 10 mg ml^−1^ driselase or 1 mg ml^−1^ chitinase produced 2.6 × 10^7^ protoplasts ml^−1^ or 7.6 × 10^6^ protoplasts ml^−1^, respectively (Shimizu et al., 1989). In addition, a study by Ranga et al. showed that 3.0 × 10^7^ protoplasts ml^−1^ could be obtained by incubating *Metarhizium anisopliae* mycelia with 9 mg ml^−1^ lysing enzyme for 3 h (Ranga and Saini, 2011). However, mixed enzymes usually yield better results than single enzymes. For example, incubating *M*. *anisopliae* mycelia with 6 mg ml^−1^ driselase, novozyme and zymolyase for 3 h yielded 1.2 × 10^8^ protoplasts ml^−1^ (Shi and Shimizu, 1996). Therefore, in this study, the protoplasts of *C*. *javanica* were treated with mixed enzymes (3 mg ml^−1^ lysing enzyme, 7 mg ml^−1^ driselase, and 0.5 mg ml^−1^ chitinase), and protoplasts were obtained at a concentration of 1.31 × 10^7^ protoplasts ml^−1^, which was sufficient for use in subsequent experiments.

Protoplast uptake of exogenous DNA is achieved by incubating protoplasts with DNA followed by incubation with a solution containing CaCl_2_ and PEG (Liu and Friesen, 2012; Amalamol et al., 2022). The molecular weight and concentration of PEG affect the conversion efficiency (Li et al., 2017; Amalamol et al., 2022). Previous studies have shown that low-molecular-weight PEG has a better effect than high-molecular-weight PEG, and most results indicate that high concentrations of PEG have a better effect than low concentrations of PEG (Becker and Lundblad, 1994; Li et al., 2017). We found that, consistent with existing reports, the conversion efficiency of PEG2000 (low molecular weight) was higher than that of PEG4000 and PEG6000 (high molecular weight). One difference in our study was that a lower concentration of PEG2000 (20%) resulted in a higher conversion efficiency; this difference may be related to interspecies differences. Therefore, we chose 20% PEG2000 as the PEG molecular weight and concentration for the transformation of *C*. *javanica* BE01. The amount of DNA in the transformation reaction is another factor that affects transformation efficiency (Amalamol et al., 2022). However, there are differences in the relationship between DNA quantity and transformation efficiency among different species. Díaz et al. (2019) found that 3 μg of DNA transformed *Pseudogymnoascus verrucosus* more efficiently than 5 μg or 10 μg of DNA. In contrast, Wang et al. (2018) showed that the transformation efficiency of *Gaeumannomyces tritici* with 6 μg of DNA was higher than that with 4 μg or 8 μg of DNA. Our results showed that the transformation efficiency of *C*. *javanica* BE01 was higher with 5 μg of DNA than with 2 μg or 10 μg of DNA. Following PEG treatment, the protoplast mixture usually needs to recover in regeneration medium before the protoplast mixture is cultured on selective medium (Liu and Friesen, 2012). The optimum recovery time also varies among species. It has been reported that *Pleurotus ostreatus* protoplasts need to recover for 18–24 h before they can be cultured on selective media, while *P*. *verrucosus* protoplasts must be allowed to recover for 24 h before transformants can be obtained (Li et al., 2006; Díaz et al., 2019). Our results showed that the protoplast mixture could be cultured on selective medium with the highest transformation efficiency after 6 h of recovery.

Proteases secreted by EPFs are considered key virulence factors that allow fungi to penetrate the insect epidermis (Wang et al., 2013). Among the enzymes secreted during fungal invasion, subtilisin-like Pr1 proteases have attracted considerable attention over the past few decades because of their potential roles in causing fungal disease and death in the host (Gao et al., 2020). The protease Pr1A, which is widely recognized to play an important role in degrading insect cuticles, has been suggested as a possible candidate for the development of advanced engineered biopesticides (Zhang et al., 2008). In this study, we overexpressed *CJPRB* (*Pr1A*) through the abovementioned PEG-mediated protoplast transformation system, which enhanced the virulence of *C*. *javanica* BE01, shortening its LT_50_ by 25.28% compared with that of the WT strain. Similar to our results, the LT_50_ of *Pr1A*-overexpressing *M*. *anisopliae* against *Manduca sexta* was 25% shorter than that of the WT strain (St Leger et al., 1996). However, the LT_50_ of *B*. *bassiana* against *Myzus persicae* was only 11% shorter than that of the WT strain (Fan et al., 2010). The above results clearly show that overexpressing *CJPRB* in *C*. *javanica* BE01 shortens the LT_50_ significantly more than overexpressing Pr1A in *B*. *bassiana*. In addition, another subtilisin-like protease of this family, Pr1H, may also play a role in infection by fungal pathogens. Wang et al. (2013) found that the *Pr1H* gene of *C*. *farinosa* was upregulated 11-fold after induction for 12 h in medium supplemented with cuticle material. Other studies have shown that *Pr1H* is activated 6 h after *M*. *anisopliae* infection of *Diaphorina citri* and remains active until 144 h of infection (Rosas-García et al., 2018). Interestingly, Pr1A is not activated during the entire infection process (Rosas-García et al., 2018). The above results indicate that the Pr1H protease may play a certain role in the infection process of EPFs. Therefore, we overexpressed *CJPRB1* (*Pr1H*) in *C*. *javanica* BE01 and found that the virulence of the strain was enhanced, as its LT_50_ increased by 54.78% compared with that of the WT strain. The above results indicate that there are differences in the expression pattern and degree of action of Pr1 proteins among different species.

In addition to the important role of the Pr1 protease in fungal infection, recent studies have shown that the acidic environmental hydrolase tripeptidyl peptidase (CLN2), belonging to the S53serine protease family, which is widely present in entomopathogens of *Cordycipitaceae*, plays a role in its pathogenicity in insects (the hemolymph of most insects is weakly acidic) (Wyatt et al., 1956; Stumpf et al., 2017; Lin et al., 2019). Studies have shown that *CLN2* is upregulated by more than 15,000-fold after 48 h of *C*. *javanica* IJ1G infection; *CLN2* also tends to be upregulated during *B*. *bassiana* infection (Tartar and Boucias, 2004; Lin et al., 2019). Our results showed that the LT_50_ of the *CJCLN2-1* overexpressing strain against *H*. *cunea* was shortened by 53.25% compared with that of the WT strain, indicating that the CJCLN2-1 protease dose impacts the fungal infection process.

In our study, strains overexpressing *CJPRB*, *CJPRB1*, and *CJCLN2-1* were not significantly different from the WT strain in terms of colony growth, conidial yield or conidial germination rate. Our results showed that the mortality of the *CJPRB1*-overexpressing strain was significantly higher than that of the WT strain at 4–5 days after infection. This may be because *CJPRB1* overexpression enhanced the efficiency of fungal penetration into the insect cuticle and accelerated the fungal infection rate. Similarly, *Lecanicillium lecanii* expressing the *Pr1A* gene from *B*. *bassiana* showed no significant differences from the WT strain in colony growth, conidial yield, and conidial germination rate (Zhang et al., 2016). The *Pr1A*-expressing strain exhibited a lower survival rate for *Aphis gossypii* within 3–5.5 days of infestation than the WT strain (Zhang et al., 2016). Moreover, studies have shown that the Pr1 protease can degrade insect immunity proteins and detoxification proteins in the hemolymph, thereby reducing insect immunity and accelerating fungal infection (Gillespie et al., 2000). The mortality of *H*. *cunea* caused by overexpression of the *CJCLN2-1* strain was higher than that caused by the WT strain between 4 and 10 days of infection, indicating that CJCLN2-1 protease may play a long-term role in fungal infection. Lin et al. also found that *CLN2* was upregulated throughout the *C*. *javanica* IJ1G infection process (Lin et al., 2019).

In conclusion, our study is the first to report the genetic transformation system of *C*. *javanica*, providing technical support for subsequent research on virulence-related genes. In addition, we confirmed by an insect biology assay that the subtilisin-like proteases CJPRB and CJPRB1 and tripeptidyl peptidase CJCLN2-1 contribute to the virulence of *C*. *javanica*, specifically by shortening the LT_50_ of *H*. *cunea* larvae. This provides a theoretical basis for the subsequent targeted induction of related protein expression to further improve the virulence of *C*. *javanica*. However, there remain limitations in the use of transgenic strains. The safety risks of the use of transgenic EPFs are associated mainly with ecological effects. These mainly include nontarget effects, gene drift potential, ecological adaptability, adaptability to environmental stress and diffusion capability. In China, before commercialization, genetically modified microbial preparations generally have to go through four stages of experimental research, intermediate testing, environmental release, and safety production testing and can only undergo a variety of verification after the researchers apply for agricultural genetically modified organism safety certificates. Therefore, there remain mainly hurdles before the commercialization of transgenic strains.

## Data availability statement

The datasets presented in this study can be found in online repositories. The names of the repository/repositories and accession number(s) can be found at: https://www.ncbi.nlm.nih.gov/genbank/, OM468894, OM468895, and OM468896.

## Author contributions

WW participated in the study design, acquisition of data, and analysis and interpretation of data. YW participated in the acquisition of data. GD and FC supervised the experiments and critically read the manuscript. All authors contributed to the article and approved the submitted version.

## Funding

This study was supported by grants from the National Key Research and Development Program of China (2017YFD0600104).

## Conflict of interest

The authors declare that the research was conducted in the absence of any commercial or financial relationships that could be construed as a potential conflict of interest.

## Publisher’s note

All claims expressed in this article are solely those of the authors and do not necessarily represent those of their affiliated organizations, or those of the publisher, the editors and the reviewers. Any product that may be evaluated in this article, or claim that may be made by its manufacturer, is not guaranteed or endorsed by the publisher.

## References

[ref1] Amalamol D. Ashwin N. M. R. Lakshana K. V. Nirmal Bharathi M. Ramesh Sundar A. Sukumaran R. K.. (2022). A highly efficient stratagem for protoplast isolation and genetic transformation in filamentous fungus *Colletotrichum falcatum*. Folia Microbiol. 67, 479–490. doi: 10.1007/s12223-022-00950-z 35106705

[ref2] Becker D. M. Lundblad V. (1994). Introduction of DNA into yeast cells. Curr. Protoc. Mol. Biol. 27:13.7.1-13.7.10. doi: 10.1002/0471142727.mb1307s27 18265102

[ref3] Cabanillas H. E. Jones W. A. (2009). Effects of temperature and culture media on vegetative growth of an entomopathogenic fungus *Isaria* sp. (Hypocreales: Clavicipitaceae) naturally affecting the whitefly, *Bemisia tabaci* in Texas. Mycopathologia 167, 263–271. doi: 10.1007/s11046-008-9176-2, PMID: 19125352

[ref4] Campbell E. I. Kinghorn J. R. Kana'n G. J. M. Unkles S. E. Panter C. (2002). Genetic transformation of the mosquito pathogenic fungus *Culicinomyces clavisporus*. Biocontrol Sci. Tech. 12, 395–399. doi: 10.1080/09583150220128176

[ref5] Chen M. Zhang D. M. Peng F. Li Z. Z. (2014). Wettable powder development of Isaria javanica for control of the lesser green leafhopper *Empoasca vitis*. Chin. J. Biol. Control 30, 51–57. doi: 10.16409/j.cnki.2095-039x.2014.01.013

[ref6] Díaz A. Villanueva P. Oliva V. Gil-Durán C. Fierro F. Chávez R.. (2019). Genetic transformation of the filamentous fungus *Pseudogymnoascus verrucosus* of antarctic origin. Front. Microbiol. 10:2675. doi: 10.3389/fmicb.2019.02675, PMID: 31824460 PMC6883257

[ref7] Fan Y. Pei X. Guo S. Zhang Y. Luo Z. Liao X.. (2010). Increased virulence using engineered protease-chitin binding domain hybrid expressed in the entomopathogenic fungus *Beauveria bassiana*. Microb. Pathog. 49, 376–380. doi: 10.1016/j.micpath.2010.06.013, PMID: 20674735

[ref8] Fang W. Feng J. Fan Y. Zhang Y. Bidochka M. J. Leger R. J.. (2009). Expressing a fusion protein with protease and chitinase activities increases the virulence of the insect pathogen *Beauveria bassiana*. J. Invertebr. Pathol. 102, 155–159. doi: 10.1016/j.jip.2009.07.013, PMID: 19666027

[ref9] Gallou A. Serna Domínguez M. G. Berlanga Padilla A. M. Ayala Zermeño M. A. Mellín Rosas M. A. Montesinos Matías R.. (2016). Species clarification of *Isaria* isolates used as biocontrol agents against *Diaphorina citri* (Hemiptera: Liviidae) in Mexico. Fungal Biol. 120, 414–423. doi: 10.1016/j.funbio.2015.11.009, PMID: 26895870

[ref10] Gao B. J. Mou Y. N. Tong S. M. Ying S. H. Feng M. G. (2020). Subtilisin-like Pr1 proteases marking the evolution of pathogenicity in a wide-spectrum insect-pathogenic fungus. Virulence 11, 365–380. doi: 10.1080/21505594.2020.1749487, PMID: 32253991 PMC7199741

[ref11] Gillespie J. P. Bailey A. M. Cobb B. Vilcinskas A. (2000). Fungi as elicitors of insect immune responses. Arch. Insect Biochem. Physiol. 44, 49–68. doi: 10.1002/1520-6327(200006)44:2<49::AID-ARCH1>3.0.CO;2-F 10861866

[ref12] He L. Guo W. Li J. Meng Y. Wang Y. Lou H.. (2020). Two dominant selectable markers for genetic manipulation in *Neurospora crassa*. Curr. Genet. 66, 835–847. doi: 10.1007/s00294-020-01063-1, PMID: 32152733

[ref13] Hu X. Y. (2014). Assessing the potential of entomopathogenic isaria javanica for management of the greenhouse insect pests. China, M.D: National Taiwan University.

[ref14] Huang J. Wang A. Huang C. Sun Y. Song B. Zhou R.. (2020). Generation of marker-free pbd-2 knock-in pigs using the crispr/cas9 and cre/loxp systems. Genes 11:951. doi: 10.3390/genes11080951, PMID: 32824735 PMC7465224

[ref15] Huang Z. Hao Y. F. Gao T. N. Huang Y. Ren S. X. Keyhani N. O. (2016). The *Ifchit1* chitinase gene acts as a critical virulence factor in the insect pathogenic fungus *Isaria fumosorosea*. Appl. Microbiol. Biotechnol. 100, 5491–5503. doi: 10.1007/s00253-016-7308-z, PMID: 26910039

[ref16] Itaya A. Zheng S. Simmonds D. (2018). Establishment of neomycin phosphotransferase II (nptII) selection for transformation of soybean somatic embryogenic cultures. *In Vitro* Cell. Dev. Biol. – plant 54, 184–194. doi: 10.1007/s11627-017-9875-9

[ref17] Kepler R. M. Luangsa-ard J. J. Hywel-Jones N. L. Quandt C. A. Sung G.-H. Rehner S. A.. (2017). A phylogenetically-based nomenclature for Cordycipitaceae (Hypocreales). IMA Fungus 8, 335–353. doi: 10.5598/imafungus.2017.08.02.08, PMID: 29242779 PMC5729716

[ref18] Li D. Tang Y. Lin J. Cai W. (2017). Methods for genetic transformation of filamentous fungi. Microb. Cell Factories 16:168. doi: 10.1186/s12934-017-0785-7, PMID: 28974205 PMC5627406

[ref19] Li G. Li R. Liu Q. Wang Q. Chen M. Li B. (2006). A highly efficient polyethylene glycol-mediated transformation method for mushrooms. FEMS Microbiol. Lett. 256, 203–208. doi: 10.1111/j.1574-6968.2006.00110.x, PMID: 16499607

[ref20] Lim F. H. Rasid O. A. Idris A. S. As'wad A. W. M. Vadamalai G. Parveez G. K. A.. (2021). Enhanced polyethylene glycol (PEG)-mediated protoplast transformation system for the phytopathogenic fungus Ganoderma boninense. Folia Microbiol. 66, 677–688. doi: 10.1007/s12223-021-00852-6, PMID: 34041694

[ref21] Lin R. M. Zhang X. Xin B. Zou M. L. Gao Y. Y. Qin F. F.. (2019). Genome sequence of *Isaria javanica* and comparative genome analysis insights into family S53 peptidase evolution in fungal entomopathogens. Appl. Microbiol. Biotechnol. 103, 7111–7128. doi: 10.1007/s00253-019-09997-4, PMID: 31273397

[ref22] Liu X. Xia Y. Zhang Y. Liang L. Xiong Z. Wang G.. (2021). Enhancement of antroquinonol production via the overexpression of 4-hydroxybenzoate polyprenyl transferase biosynthesis-related genes in Antrodia cinnamomea. Phytochemistry 184:112677. doi: 10.1016/j.phytochem.2021.112677, PMID: 33556840

[ref23] Liu Z. Friesen T. L. (2012). Polyethylene glycol (PEG)-mediated transformation in filamentous fungal pathogens. Methods Mol. Biol. 835, 365–375. doi: 10.1007/978-1-61779-501-5_21, PMID: 22183664

[ref24] Lou H.-W. Ye Z.-W. Yu Y.-H. Lin J.-F. Guo L.-Q. Chen B.-X.. (2019). The efficient genetic transformation of *Cordyceps militaris* by using mononuclear protoplasts. Sci. Hortic. 243, 307–313. doi: 10.1016/j.scienta.2018.08.043

[ref25] Nai Y. S. Lee M. R. Kim S. Lee S. J. Kim J. C. Yang Y. T.. (2017). Relationship between expression level of hygromycin B-resistant gene and agrobacterium tumefaciens-mediated transformation efficiency in *Beauveria bassiana* JEF-007. J. Appl. Microbiol. 123, 724–731. doi: 10.1111/jam.13529, PMID: 28667709

[ref26] Qu S. Wang S. B. (2018). Interaction of entomopathogenic fungi with the host immune system. Dev. Comp. Immunol. 83, 96–103. doi: 10.1016/j.dci.2018.01.010, PMID: 29355579

[ref27] Ranga V. Saini G. K. (2011). Biochemical and molecular characterization of wild-type and fused protoplasts of *Beauveria bassiana* and *Metarhizium anisopliae*. Folia Microbiol. 56, 289–295. doi: 10.1007/s12223-011-0055-8, PMID: 21818611

[ref28] Ren S. X. Hu Q. B. (2009). The strain of Isaria javanica and its application in the control of Spodoptera litura. China Patent CN101514325. Beijing: China National Intellectual Property Administration.

[ref29] Rosas-García N. M. López-Barrera G. L. Mireles-Martínez M. Villegas-Mendoza J. M. (2018). Activación de las isoformas del gen pr1 de *Metarhizium anisopliae* 1 durante la patogénesis en *Diaphorina citri*. Southwest. Entomol. 43, 199–207. doi: 10.3958/059.043.0112

[ref30] Schrank A. Vainstein M. H. (2010). *Metarhizium anisopliae* enzymes and toxins. Toxicon 56, 1267–1274. doi: 10.1016/j.toxicon.2010.03.008 20298710

[ref31] Shao C. Yin Y. Qi Z. Li R. Song Z.. (2015). Agrobacterium tumefaciens-mediated transformation of the entomopathogenic fungus Nomuraea rileyi. Fungal Genet. Biol. 83, 19–25. doi: 10.1016/j.fgb.2015.08.002, PMID: 26275508

[ref32] Shi L. Shimizu S. (1996). Isolation of protoplasts from *Metarhizium anisopliae* var. majus. Nihon sanshigaku zasshi 65, 201–204. doi: 10.11416/kontyushigen1930.65.201

[ref33] Shimazu M. Takatsuka J. (2010). *Isaria javanica* (anamorphic Cordycipitaceae) isolated from gypsy moth larvae, *Lymantria dispar* (Lepidoptera: Lymantriidae) in Japan. Appl. Entomol. Zool. 45, 497–504. doi: 10.1303/aez.2010.497

[ref34] Shimizu S. Katou M. Matsumot T. Kurisu K. (1989). Isolation and reversion of protoplasts from an entomopathogenic fungus, *Paecilomyces fumosoroseus*. Nihon Ōyō Dōbutsu Konchū Gakkai shi 33, 47–50. doi: 10.1303/jjaez.33.47

[ref35] St Leger R. Joshi L. Bidochka M. J. Roberts D. W. (1996). Construction of an improved mycoinsecticide overexpressing a toxic protease. Proc. Natl. Acad. Sci. U. S. A. 93, 6349–6354. doi: 10.1073/pnas.93.13.6349, PMID: 8692818 PMC39025

[ref36] Stumpf M. Muller R. Gassen B. Wehrstedt R. Fey P. Karow M. A.. (2017). A tripeptidyl peptidase 1 is a binding partner of the Golgi pH regulator (GPHR) in *Dictyostelium*. Dis. Model. Mech. 10, 897–907. doi: 10.1242/dmm.029280, PMID: 28546289 PMC5536908

[ref37] Tartar A. Boucias D. G. (2004). A pilot-scale expressed sequence tag analysis of *Beauveria bassiana* gene expression reveals a tripeptidyl peptidase that is differentially expressed in vivo. Mycopathologia 158, 201–209. doi: 10.1023/B:MYCO.0000041905.17948.42, PMID: 15518349

[ref38] Thomas M. B. Read A. F. (2007). Can fungal biopesticides control malaria? Nat. Rev. Microbiol. 5, 377–383. doi: 10.1038/nrmicro1638 17426726

[ref39] Valero-Jiménez C. A. Debets A. J. M. van Kan J. A. L. Schoustra S. E. Takken W. Zwaan B. J.. (2014). Natural variation in virulence of the entomopathogenic fungus *Beauveria bassiana* against malaria mosquitoes. Malar. J. 13:479. doi: 10.1186/1475-2875-13-479, PMID: 25480526 PMC4364330

[ref40] Vicente R. Perez P. Martinez-Carrasco R. Usadel B. Kostadinova S. Morcuende R. (2015). Quantitative RT-PCR platform to measure transcript levels of c and n metabolism-related genes in durum wheat: transcript profiles in elevated [Co_2_] and high temperature at different levels of n supply. Plant Cell Physiol. 56, 1556–1573. doi: 10.1093/pcp/pcv079, PMID: 26063390

[ref41] Wang M. Zhang J. Wang L. Han L. Zhang X. Feng J. (2018). Optimization of production conditions for protoplasts and polyethylene glycol-mediated transformation of *Gaeumannomyces tritici*. Molecules 23:1253. doi: 10.3390/molecules23061253, PMID: 29794975 PMC6100196

[ref42] Wang W. X. Zhou L. F. Dong G. P. Chen F. M. (2019). Isolation and identification of entomopathogenic fungi and an evaluation of their actions against the larvae of the fall webworm, *Hyphantria cunea* (Drury) (Lepidoptera: Arctiidae). BioControl 65, 101–111. doi: 10.1007/s10526-019-09982-w

[ref43] Wang Z. Meng H. Zhuang Z. Chen M. Xie L. Huang B. (2013). Molecular cloning of a novel subtilisin-like protease (Pr1A) gene from the biocontrol fungus *Isaria farinosa*. Appl. Entomol. Zool. 48, 477–487. doi: 10.1007/s13355-013-0208-0

[ref44] Wei G. Lai Y. Wang G. Chen H. Li F. Wang S. (2017). Insect pathogenic fungus interacts with the gut microbiota to accelerate mosquito mortality. Proc. Natl. Acad. Sci. U. S. A. 114, 5994–5999. doi: 10.1073/pnas.1703546114, PMID: 28533370 PMC5468619

[ref45] Wei Y. Zhou X. Liu L. Lu J. Wang Z. Yu G.. (2010). An efficient transformation system of taxol-producing endophytic fungus EFY-21 (*Ozonium* sp.). Afr. J. Biotechnol. 9, 1726–1733. doi: 10.5897/AJB2010.000-3019

[ref46] Wyatt G. R. Loughheed T. C. Wyatt S. S. (1956). The chemistry of insect hemolymph: organic components of the hemolymph of the silkworm, *bombyx mori*, and two other species. J. Gen. Physiol. 39, 853–868. doi: 10.1085/jgp.39.6.853, PMID: 13346040 PMC2147577

[ref47] Zhang Q. Zhao L. Shen M. Liu J. Li Y. Xu S.. (2022). Establishment of an efficient polyethylene glycol (peg)-mediated transformation system in *pleurotus eryngii* var. ferulae using comprehensive optimization and multiple endogenous promoters. J. Fungi 8:186. doi: 10.3390/jof8020186, PMID: 35205941 PMC8876744

[ref48] Zhang W. Yueqing C. Yuxian X. (2008). Cloning of the subtilisin Pr1A gene from a strain of locust specific fungus, *Metarhizium anisopliae*, and functional expression of the protein in *Pichia pastoris*. World J. Microbiol. Biotechnol. 24, 2481–2488. doi: 10.1007/s11274-008-9771-x

[ref49] Zhang Y. J. Xie M. Zhang X. L. Peng D. L. Yu W. B. Li Q.. (2016). Establishment of polyethylene-glycol-mediated protoplast transformation for *Lecanicillium lecanii* and development of virulence-enhanced strains against *Aphis gossypii*. Pest Manag. Sci. 72, 1951–1958. doi: 10.1002/ps.4236, PMID: 26800336

