# Supplementary material for: Development of Cordyceps javanica BE01 with enhanced virulence against Hyphantria cunea using polyethylene glycol-mediated protoplast transformation
Source: Front Microbiol. 2022 Sep 2;13:972425. doi: 10.3389/fmicb.2022.972425 (PMC9478556; doi:10.3389/fmicb.2022.972425)
Supplement: Supplementary file 1 [file Data_Sheet_1.docx]

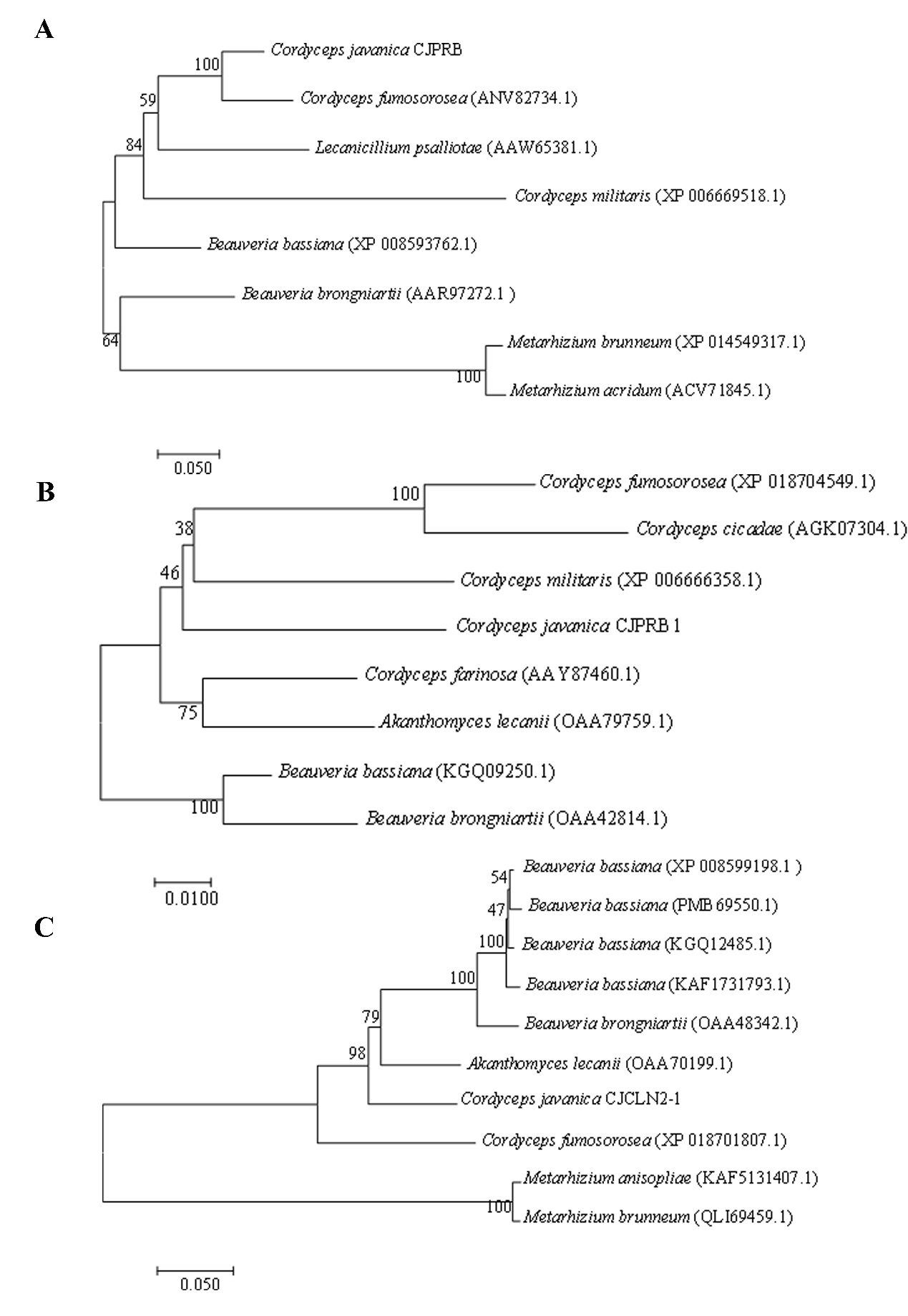


Figure S1 *Cordyceps javanica* protease phylogenetic analyses (Neighbour-joining tree). A Alignment of the amino acid sequences of subtilisin-like protease from different organisms sharing homology to CJPRB (PR1A). B Alignment of the amino acid sequences of subtilisin-like protease from different organisms sharing homology to CJPRB1(PR1H). C Alignment of the amino acid sequences of tripeptidyl peptidase from different organisms sharing homology to CJCLN2-1. Branch termini are tagged in light of isolate species and GenBank accession numbers. The numbers above (or below) the nodes show the bootstrap values occurring after 1,000 replications. Scale bars represent the average number of nucleotide substitutions per site (MEGA version 7.0).

Table S1 Identity of homologous proteases with subtilisin-like proteases CJPRB and the CJPRB1 and the tripeptidyl peptidase CJCLN2-1

| Species | Identity |
| --- | --- |
| CJPRB | |
| *Cordyceps fumosorosea* (ANV82734.1) | 90.65% |
| *Lecanicillium psalliotae* (AAW65381.1) | 81.02% |
| *Cordyceps militaris* (XP 006669518.1) | 66.10% |
| *Beauveria bassiana* (XP 008593762.1) | 81.14% |
| *Beauveria brongniartii* (AAR97272.1) | 77.21% |
| *Metarhizium brunneum* (XP 014549317.1) | 65.14% |
| *Metarhizium acridum* (ACV71845.1) | 65.43% |
| CJPRB1 | |
| *Cordyceps fumosorosea* (XP 018704549.1) | 89.49% |
| *Cordyceps cicadae* (AGK07304.1) | 87.74% |
| *Cordyceps militaris* (XP 006666358.1) | 91.05% |
| *Cordyceps farinosa* (AAY87460.1) | 91.83% |
| *Akanthomyces lecanii* (OAA79759.1) | 92.02% |
| *Beauveria bassiana* (KGQ09250.1) | 90.66% |
| *Beauveria brongniartii* (OAA42814.1) | 89.69% |
| CJCLN2-1 | |
| *Cordyceps fumosorosea* (XP 018701807.1) | 80.53% |
| *Akanthomyces lecanii* (OAA70199.1) | 88.28% |
| *Beauveria bassiana* (XP 008599198.1) | 84.98% |
| *Beauveria bassiana* (PMB69550.1) | 84.65% |
| *Beauveria bassiana* (KGQ12485.1) | 85.48% |
| *Beauveria bassiana* (KAF1731793.1) | 84.82% |
| *Beauveria brongniartii* (OAA48342.1) | 83.66% |
| *Metarhizium brunneum* (QL169459.1) | 59.05% |
| *Metarhizium anisopliae* (KAF5131407.1) | 59.71% |

Table S2 Oligonucleotide primers used for PCR amplification and qRT-PCR test and

| Primer | Sequence (5′–3′) |
| --- | --- |
| CJPRB-F | TTTCGTAGGAACCCAATCTTCAAAATGGCCCCCGTTGTTGAG |
| CJPRB-R | CACCACCCCGGTGAACAGCTCCTCGCCCTTGCTCACCTAAGTGGCGCCGTTGAAGGC |
| CJPRB1-F | TTTCGTAGGAACCCAATCTTCAAAATGTCGTCCTTCAAGATTG |
| CJPRB1-R | CACCACCCCGGTGAACAGCTCCTCGCCCTTGCTCACTTACTCCATGTGGGAGAGG |
| CJCLN2-1-F | TTTCGTAGGAACCCAATCTTCAAAATGATCCCTCTTGGTGACC |
| CJCLN2-1-R | CACCACCCCGGTGAACAGCTCCTCGCCCTTGCTCACTTACAAGTCCTTGAAGTAAGC |
| RT-qPCR Primers | Sequence (5′–3′) |
| EF-1F | TCCGTCAAGGAAATCCGTCGTG |
| EF-1R | ACGCACATGGGCTTGGAGAG |
| CJPRBF | ATTGGCTCCAAGAAGTGGGG |
| CJPRBR | CGTTTTCCTATCCGAGGCGA |
| CJPRB1F | GGTGCCTCCACCATTGATGA |
| CJPRB1R | GTAGTAGGCGAGAAGACCGC |
| CJCLN2-1F | TGGCACATTCAAGCCTACT |
| CJCLN2-1R | CTTCGCCGTTCTTTCCCT |

verification of fungal transformants
